# Reducing Respiratory Complications During Electroconvulsive Therapy (ECT) With Smaller Doses of Succinylcholine in a Morbidly Obese Patient: A Case Report

**DOI:** 10.7759/cureus.65654

**Published:** 2024-07-29

**Authors:** Rensheng V Zhang, Brent R Carr

**Affiliations:** 1 Department of Anesthesiology, University of Florida College of Medicine, Gainesville, USA; 2 Department of Psychiatry, University of Florida College of Medicine, Gainesville, USA

**Keywords:** upper airway obstruction, succinylcholine, severe hypoxia, prolonged paralysis, morbid obesity, electroconvulsive therapy (ect)

## Abstract

Anesthesia for electroconvulsive therapy (ECT) requires proper medications and airway management. Besides an induction agent such as methohexital, a neuromuscular blocker such as succinylcholine (SCh) is often given for muscle relaxation. To maintain the patient’s oxygen saturation, mask ventilation is required due to this transient chemical paralysis even in the presence of adequate preoxygenation. A morbidly obese, middle-aged female experienced multiple life-threatening hypoxic episodes due to “bronchospasms” during prior ECT treatments. A drastic reduction in the SCh dose to about half of the original dose led to much smoother anesthesia courses with no more hypoxic episodes during subsequent ECT treatments. We believe that the lower dosing of SCh avoided a long period of chemical paralysis, which led to a quick return of spontaneous respiration, shortened the need for airway support, and therefore avoided hypoxic episodes in subsequent ECT treatments.

## Introduction

Electroconvulsive therapy (ECT) is a well-established, safe, and highly efficacious treatment modality [1]. General anesthesia, often with mask ventilation for airway support, fundamentally improves treatment safety and tolerability [2]. Anesthesia provides amnesia and paralysis, which are often achieved with proper dosing of methohexital and succinylcholine (SCh), respectively, although other drugs can be used as alternatives. An insufficient dose of methohexital may not provide sufficient amnesia, while supraoptimal dosing prolongs anesthesia time, interferes with seizure duration and quality, and complicates recovery. Similarly, the neuromuscular blockade must be adequate to mitigate harmful muscle contractions during an ECT-induced tonic-clonic seizure; excess SCh dosing prolongs paralysis and delays recovery of muscle tone necessary for spontaneous breathing and airway patency [3]. A wide range of SCh dosage has been used, although some authors recommend a SCh dose of 0.9 mg/kg based on total body weight (TBW) [4]. A higher SCh dose of 1 mg/kg provided better seizure movement modification than a lower dose of 0.5 mg/kg, although the higher dosing also caused a small delay in recovery time from respiratory paralysis [5]. These studies provided no information for morbidly obese patients on SCh dosing during ECT, placing them at higher risk during this relatively safe procedure. 

This article was previously presented as an abstract at the 5th International Brain Stimulation Conference on February 18-22, 2023.

## Case presentation

Our patient was a 48-year-old female diagnosed with schizoaffective disorder, bipolar type, with a 20-year deteriorating medication refractory course that responded briskly to an initial series of thrice weekly ECTs. Recurrence of symptoms led to a need for subsequent ECT treatments, also with good response. Her medical history was also significant for morbid obesity, obstructive sleep apnea, and smoking. Her BMI was 43 with a height of 160 cm and weight of 110 kg. Her examination showed a typical difficult airway with a small mouth opening, Mallampati score of III, thyromental distance of less than 3 finger widths, and limited neck extension.

The ECT treatment was done in a high-volume setting without an anesthesia machine. A Mapleson breathing circuit was used for assisted ventilation with 100% oxygen. The patient received a total of 45 ECTs over three years. In the first nine months, she received 17 ECTs under the care of seven anesthesiologists. Severe hypoxia occurred during four ECTs (2nd, 14th, 16th, and 17th), and epinephrine was given intravenously (10 to 20 mcg) or subcutaneously (0.3 mg) for treatment of presumed bronchospasm. Anesthesia was provided by three anesthesiologists who cared for 12 of the 17 ECTs. The medications given included intravenous methohexital or ketamine, plus 100 to 120 mg of intravenous SCh. The SCh doses were about 1 mg/kg TBW. Additional airway maneuvers during these four ECTs included placement of a nasal trumpet or oral airway. More advanced airway support such as endotracheal intubation or laryngeal mask airway was not required.

The author’s (RVZ) first encounter with the patient was for the 18th ECT. Prior hypoxia episodes were very concerning, leading to a comprehensive pre-anesthesia evaluation. The patient had no history of asthma or chronic obstructive pulmonary disease and had no problems during previous general anesthesia for hemorrhoidectomy and appendectomy. The most likely cause of her severe hypoxia during those four ECTs was inadequate ventilation due to her obstructed airway with prolonged paralysis from SCh. The decision was made to proceed with her 18th ECT. She was given 90 mg methohexital and 60 mg SCh. This SCh dose, correlating to 0.5 mg/kg TBW or 1 mg/kg ideal body weight (IBW), was about half of the doses given during those four ECTs with hypoxia. The ECT treatment proceeded smoothly without the need for additional airway maneuvers such as nasal or oral airway placement. The neuromuscular blockade was adequate for seizure modification, spontaneous breathing returned within minutes, oxygen saturation remained above 96%, and anesthesia was completed within 10 minutes following ECT treatment. After this smooth anesthetic course, this low-dose SCh strategy was adopted for the subsequent 27 ECTs over the next two and a half years by 19 anesthesiologists. Anesthesia was typically induced with 60 to 100 mg methohexital and 60 to 80 mg SCh. All 27 ECTs were uneventful without severe hypoxic episodes.

The four ECTs with severe hypoxia all had very long anesthesia time, ranging from 38 to 64 minutes with a mean of 43.8 minutes. This apparently was due to prolonged paralysis from high dose SCh and required prolonged high-level ventilatory support and extra time to recover from hypoxia. In comparison, the anesthesia time from nine randomly selected ECTs with the low-dose SCh had much shorter anesthesia time, ranging from 13 to 26 minutes with a mean of 18.7 minutes. The shorter anesthesia time was also associated with quicker and smoother recovery in the postanesthesia recovery room. The patient provided written informed consent for the publication of this case report.

## Discussion

Succinylcholine is commonly used for paralysis during ECT with a very wide range of dosages, although some guidelines recommend 0.9 mg/kg [4]. There is a lack of information regarding patients with morbid obesity, placing this patient population at a higher risk for prolonged neuromuscular blockade and severe hypoxia during ECT treatments.

In an SCh dosing study, the effects of muscle relaxation and recovery time were recorded in morbidly obese patients, comparing SCh doses at 1 mg/kg based on IBW vs TBW. An average dose of 61 mg SCh was given for the IBW group vs 122 mg SCh for the TBW group. While the onset time for maximum neuromuscular blockade occurred around 90 seconds after SCh injection in both groups, the recovery time to 50% single twitch height on the TOF-Watch SX acceleromyograph was significantly longer for the TBW group (8.5 vs 5 minutes) [6]. This is consistent with the longer recovery time from respiratory paralysis in ECT patients who received larger doses of SCh [5]. The 50% single twitch height is an important concept because it represents a functional recovery of neuromuscular function that is adequate for spontaneous ventilation with FIO2 = 1.0 with a patent airway [7]. Partial paralysis is also associated with inspiratory upper airway collapse [3], leading to upper airway obstruction. There was also a difference in oxygen reserve in patients with different physical statuses. After proper preoxygenation, oxygen saturation remained above 90% for only three minutes in an obese patient during apnea, compared with eight minutes in a nonobese patient [7]. Based on these studies, patients with morbid obesity are prone to desaturation during ECT.

The SCh dose given to our patient during the noted four ECTs was excessive, leading to prolonged paralysis with a collapsed upper airway. Mask ventilation was ineffective due to the patient’s intrinsic difficult airway. The presence of a poor oxygen reserve made the patient highly vulnerable to severe hypoxia during ECT treatments.

Airway management was much easier when smaller SCh doses were given. Significantly shorter paralysis and quicker return of spontaneous breathing led to a less obstructive airway and shorter dependency on assisted ventilation. It is important to note that the seizure modification was adequate when 60 mg of SCh was given to this patient because no significant body movement was noted during ECT-induced seizures. Therefore, more than 60 mg of SCh in this patient would only prolong paralysis, leading to higher risk of hypoxia without the benefit of better seizure modification.

Pseudocholinesterase (PChE) deficiency, which makes the patient more sensitive to SCh with prolonged paralysis, can be a genetic disorder or an acquired condition. Besides more than 60 genetic variants, the most significant one being atypical PChE deficiency, low PChE can be seen in many diseases or various clinical conditions. In a five-year study on 193 ECT patients, 4.7% had low PChE, and there is no correlation with age, gender, weight, or diagnosis [8]. Another five-year study of 500 ECT patients on SCh dose for neuromuscular blockade showed extreme variability with 5.8% of patients requiring very high or very low doses, and the minimum required dose was 0.29 mg/kg [9]. It is reasonable to conclude that our patient has low PChE and is sensitive to SCh, and therefore a drastic reduction in SCh dose was the correct choice for her to avoid an unnecessarily prolonged paralysis and severe hypoxia during ECT treatment.

Rocuronium-sugammadex has been shown to provide good muscle relaxation during ECT treatment with quick and reliable onset and reversal [10]. However, besides the higher cost of the medications, losing intravenous access after rocuronium administration with the inability to give sugammadex for reversal is a potentially very high-risk scenario in an obese patient. The latter is one of the reasons we were content with the choice of low-dose SCh, which worked beautifully.

Although our patient benefited from smaller doses of SCh, we do not recommend it for every patient during ECT. A higher SCh dose can provide better seizure modification and is safe for most patients without significant risk of airway obstruction and hypoxia [4]. However, the situation is different for patients with morbid obesity, especially if the patient is also sensitive to SCh. We present this case report to illustrate that SCh at 1 mg/kg IBW was the correct choice for our morbidly obese patient as this avoided prolonged paralysis and its attendant risks.

## Conclusions

Patients with morbid obesity are at high risk for unnecessarily prolonged paralysis during ECT treatment when SCh is given at the dose of 1 mg/kg based on actual body weight, which can lead to prolonged paralysis, upper airway obstruction, difficult mask ventilation, and severe hypoxia. Anesthesia care is significantly safer for these patients when a much smaller dose of SCh at 1 mg/kg IBW is used. The smaller dose of SCh still provides adequate paralysis for ECT treatment, and it comes with significantly shorter paralysis and airway obstruction, quicker recovery of spontaneous ventilation, and avoidance of severe hypoxia.
